# Where are the vulnerable children? Identification and comparison of clusters of young children with health and developmental vulnerabilities across Queensland

**DOI:** 10.1371/journal.pone.0298532

**Published:** 2024-03-15

**Authors:** Wala Draidi Areed, Aiden Price, Kathryn Arnett, Kerrie Mengersen, Helen Thompson

**Affiliations:** 1 School of Mathematical Science, Centre for Data Science, Queensland University of Technology, Brisbane, Queensland, Australia; 2 Children’s Health Queensland, Brisbane, Queensland, Australia; University of Mosul, IRAQ

## Abstract

This study aimed to better understand the vulnerability of children in their first year of school, aged between 5 years 5 months and 6 years 6 months, based on five health and development domains. Identification of subgroups of children within these domains can lead to more targeted policies to reduce these vulnerabilities. The focus of this study was to determine clusters of geographical regions with high and low proportions of vulnerable children in Queensland, Australia. This was achieved by carrying out a *K*-means analysis on data from the Australian Early Development Census and the Australian Bureau of Statistics. The clusters were then compared with respect to their geographic locations and risk factor profiles. The results are made publicly available via an interactive dashboard application developed in R Shiny.

## Introduction

Internationally, there is an increasing focus on population health among integrated care organisations and health systems [1, 2]. The goal of population health methods is to enhance the overall health of a group of people. In order to do this, it is critical to recognise the requirements of various groups within the population [3–5]. An important group in the population is children.

Healthy child development improves human capabilities by allowing children to mature and participate in economic, social, and civic life [6]. Child development includes the biological, psychological, and emotional changes that occur between birth and maturity [7]. Physical, social, emotional, speech and language, and communication skills are the five critical domains of growth [8]. Children’s development in the early years from birth to five years of age is crucial since it is at this time that the foundations for health development, emotional well-being, and life success are built [9].

Increasingly many countries, including Australia, are using national progress indicators of early children’s development to track critical developmental domains in the early years [10, 11]. The Australian Early Development Census (AEDC) provides a nationwide snapshot of children’s development at the time children commence their first year of full-time school and reports scores across the five domains of growth. Each child is given a score between zero and ten for each of the AEDC domains and, using the cut-offs established as a baseline in 2009, children falling below the 10th percentile in a domain, taking into account the age differences, are categorised as ‘developmentally vulnerable’. The AEDC reveals that the proportion of children who are developmentally vulnerable, within each developmental domain, varies considerably between geographical regions across Australia. This variation exists across the smallest geographic areas defined by the AEDC, which are referred to as local communities and are often equivalent to suburbs [12]. To address inequalities in developmental vulnerabilities, further insight is needed into the factors that contribute to such variation [13]. One method to understand the variation is cluster analysis [14].

In this paper, we review research endeavours that utilized cluster analysis to examine children’s development in various contexts, offering a comprehensive overview of its contributions to understanding the different dimensions of children’s development. Cluster analysis has played a significant role in identifying groups of children exhibiting different patterns of vulnerability across AEDC domains [15]. It has also been instrumental in exploring the associations between clusters of early life risk factors and children’s development [15, 16]. Some research papers have conducted cluster randomized trials to examine the effectiveness of parenting programs in reducing children’s developmental vulnerabilities.Clustering techniques have been applied across numerous studies to explore different facets of children’s development. Reeve et al. [17] used cluster analysis to examine children’s number development, identifying three clusters based on dot enumeration and number comparison tasks. Abenavoli et al. [18] examined at-risk low-income kindergarteners, revealing unexpected patterns, including academically strong but aggressive children and another group with poor academic engagement but without disruptive behaviour issues. Richmond-Rakerd et al. [19] found a clear link between early-life challenges, such as developmental vulnerability and adolescent difficulties, and higher public service costs in adulthood, including elevated welfare dependency, crime, and poor health. Lamb et al. [20] emphasized the importance of neighborhood policies for disadvantaged communities, noting a higher prevalence of children at risk of early developmental disadvantages in lower socioeconomic areas. Quirk et al. [21] linked kindergarten well-being clusters to later academic performance. Taylor [15] examined early life risk factors and developmental vulnerability. Russell et al. [22] focused on health equity, finding links between social determinants and developmental health. Christensen et al. [23] used latent class analysis to study patterns related to children, families, schools, and communities. Brinkman et al. [10] found a “socioeconomic disadvantage” cluster linked to developmental vulnerabilities. Williamson et al. [24] explored social and emotional developmental vulnerability. Our recent study in Queensland, Australia [25] analyzed spatial structures of preschool attendance and developmental vulnerabilities. Dea et al. [26] compared early children’s development outcomes in metropolitan areas. Watts et al. [27] explored support practices in schools using AEDC data. Watkeys et al. [16] used latent class analysis to identify distinct clusters based on perinatal and familial risk exposures at birth.

Only a few studies have taken into account the spatial aspect of children’s development when conducting clustering. For example, a space-based approach using techniques like Moran’s I and local indicators of spatial association clusters was employed to investigate the clustering of areas with high and low developmental vulnerability in Greater Sydney and Canberra, two cities in Australia [28]. None of the existing papers have comprehensively studied cluster analysis for each domain of children’s development. This paper aims to fill this gap by providing a thorough analysis of children’s development in Queensland, Australia and providing a profile for each region based on socioeconomic and educational factors. Additionally, this paper offers a comparative assessment across the five domains of children’s development as well as two indicators and includes an interactive tool for visualizing finer details.

In this study, the silhouette coefficient (Section Cluster evaluation) is used to determine the number of clusters. While the elbow method is easy to implement and the calculations required are simple, the silhouette coefficient allows evaluations of clusters on multiple criteria, and hence it is more likely that the optimal number of clusters can be determined [29].

Publicly accessible data in the population AEDC domain are frequently aggregated within geographical areas [30]. In Australia, these geographical areas are typically the statistical areas defined in the Australian Statistical Geography Standard (ASGS). In the ASGS, Statistical Areas Level 1 (SA1) is the smallest defined geographical areas and aggregate to form Statistical Areas Level 2 (SA2). There are four levels of aggregation of statistical areas, SA1 through SA4. Where personal-level information is available, it is not uncommon for data on the exact location of individuals to be missing. Even if exact location data are available, privacy and confidentiality concerns prevent the publication of person-level information. Hence this study uses data aggregated at SA2 level [30].

In this study, we aimed to identify and characterise regions in Queensland in terms of high and low vulnerability across five domains of health and development, for children in their first year of full time school. We used *K*-means clustering to identify regions, such that within a cluster of SA2s making up a region, children have similar vulnerabilities for a given AEDC domain. In characterising the regions (clusters of SA2s), we consider the factors from AEDC which are publicly available at the SA2 level: attendance at preschool, Indigenous status, mother’s language, country of birth, socioeconomic status, and remoteness status. In addition to the abridged results presented in this paper, we developed a web application to make the complete set of results accessible and more easily digestible. The web application has an intuitive interface that allows users to interactively explore child development vulnerability across the AEDC domains and across the SA2 areas of Queensland. The results of this research will support targeted early intervention strategies which can allow children to reach their maximum developmental potential.

## Materials and methods

### Case study and sources of data

Child development vulnerability data were obtained from the 2018 AEDC. The AEDC is conducted every three years and collects data on children in their first year of full-time school. The AEDC recently took place in 2021, but the most recent data available is for the 2018 census. The census is performed by classroom teachers in the child’s first year of full-time schooling across the Australian government and non-government schools, and data are collected with the agreement of parents [12]. The data provided on a child by their teacher, based on the teacher’s knowledge and observations of the child, is used to assign the child a score (0 to 10) for each AEDC developmental domain. For each domain, the child is then classified as *vulnerable* if their score is in the lowest 10% of scores for that domain using the cut-offs established as a baseline in 2009. Approximately 65,000 children (98.1% of eligible children) across 1,414 Queensland government, catholic and independent schools participated in the 2018 AEDC collection. The data were available as aggregated counts at the SA2 level. Among the 528 SA2s that make up Queensland, there was an average of 123 children per SA2, with a standard deviation of 100 [12].

All five domains of health and developmental vulnerability from the AEDC were considered in this study: physical health and wellbeing (Physical), social competence (Social), emotional maturity (Emotional), language and cognitive skills school-based (Language), and communication skills and general knowledge (Communication). We also considered two additional AEDC indicators of vulnerability: vulnerable in one or more domain/s (Vuln 1), and vulnerable in two or more domains (Vuln 2). Due to the aggregated nature of the available data, we focused on the proportion of vulnerable children within each SA2. The following data were also obtained from the 2018 AEDC for each SA2: the proportion of children who attended preschool (Preschool), the proportion who identified as Indigenous (Indigenous), the proportion with English as the mother’s language (English), the proportion with Australia as country of birth (Australia). Further data for 2018 were obtained from the Australian Bureau of Statistics (ABS) for each SA2, including the index of relative socio-economic disadvantage (IRSD) for the SA2 (1 to 10), and remoteness (Major City, Inner Regional, Outer Regional, Remote, Very Remote). The IRSD is coded from 1 (lowest) to 10 (highest) [31]; a low score suggests that the area, in general, is at a disadvantage, e.g., many low-income households, many people without qualifications or with low-skill occupations. In 2018, there were 294 major cities, 113 inner regional, 96 outer regional, 11 remote and 14 very remote SA2s in Queensland.

Between 3% and 6% of the data were missing variables in the dataset. Proportions (e.g., Preschool, Indigenous) that were missing for an SA2 were imputed using the average of the proportions from the neighbouring SA2s. For categorical data, i.e; IRSD and Remoteness, the missing value was imputed using the highest frequency category of the neighbourhood SA2s. Missing values for two islands could not be imputed, as the regions have no contiguous neighbours. As a result, the analysis carried out in this study was reduced to the remaining 526 SA2 areas.

### Clustering method

This section details the clustering method used to investigate the data clusters. All statistical analyses were conducted using R statistical software version R-4.1.3 [32]. The analyses for the *K*-means algorithm were carried out using mclust [33], and factoextra [34] packages, and the shiny package in R was used to develop the interactive dashboard [35].

#### *K*-means clustering

Cluster analysis is a mechanism for grouping (clustering) a set of objects (e.g., local communities) in such a way that objects within a group (cluster) are more similar (e.g., in terms of developmentally vulnerable) to one another than to those in other groups (clusters) [36]. There are many clustering methods: model-based versus fully empirical, parametric versus non-parametric, probabilistic versus non-probabilistic, hierarchical versus partition-based, and supervised versus unsupervised [37]. There are also many computational methods for clustering, including the expectation-maximisation (EM) algorithm [38] and a variety of simulation-based algorithms, such as Markov chain Monte Carlo (MCMC) [39]. A well-established simple non-probabilistic unsupervised partitioning method, which is employed in this study, is *K*-means clustering, where *K* denotes the number of clusters [40, 41]. Common strategies for choosing the value of *K* include the elbow method [42], gap statistic [43], silhouette coefficient [44], and canopy method [45].

The *K*-means clustering method is a popular unsupervised machine learning technique that is extensively utilised due to its simplicity and fast convergence. The *K*-means algorithm is a basic partitioning approach that utilises a distance metric for partitioning observations into clusters. The number of clusters, *K*, is determined beforehand. The centre of a cluster is known as the cluster centroid. Every data point is allocated to a cluster such that within a cluster the summed distance between the centroid and data points is minimised, and between clusters, the summed distance between cluster centroids is maximised. Some distance metrics include Euclidean distance, Manhattan distance, cosine distance, Minkowski distance and correlation distance [46]. In this study, the Euclidean distance was adopted. The chosen value of *K* directly influences both the convergence of the algorithm and the inferences. In this study, we considered a range of plausible values of *K* and chose the value that gave the best fit, as determined by the silhouette coefficient(see section Cluster evaluation).

The algorithm proceeds as follows. 1) Define the number of clusters *K*. 2) Randomly select *K* data points as the cluster centroids. 3) Assign data points to the closest cluster centroid. 4) Recompute the cluster centroids. 5) Repeat steps 3) and 4) until either the centroids do not change or the maximum number of iterations is reached [47]. In this paper, we apply the *K*-means algorithm to the proportion of vulnerable children in a SA2 for each of the five AEDC domains and two indicators.

#### Cluster evaluation

Internal and relative validation are two popular ways of evaluating a cluster analysis. Internal validation uses two fundamental principles to validate clusters: cohesion and separation. Cohesion measures the average distance between items within clusters, while separation measures the average distance of a cluster to the adjacent cluster. Clusters are confirmed in relative validation by altering the clustering algorithm’s parameters, such as the number of clusters *K*, to optimise a given measure of fit.

In this study, we adopted the silhouette method for cluster evaluation [44], which combines cohesion and separation. The similarity between the item and the cluster to which it belongs is represented by cohesion, and when compared to other clusters, it is described as separation. These comparisons may be quantified using the silhouette coefficient, which ranges from −1 to 1, with a value near 1 suggesting good identification between the item and the cluster. In general, silhouette width scores less than 0.2 or silhouette width scores greater than 0.9 are problematic; silhouette width scores of 0.5 are good, and silhouette width scores between 0.7 and 0.9 are preferable [48]. The Silhouette coefficient is given as:

$$\begin{array}{c} s\left(i\right)=\frac{b(i)-a(i)}{\text{max}\{a(i),b(i)\}}=\{\begin{array}{cc} 1-\frac{a(i)}{b(i)} & \text{if}\;a(i)<b(i)\\ 0 & \text{if}\;a(i)=b(i)\\ \frac{b(i)}{a(i)}-1 & \text{if}\;a(i)>b(i) \end{array} \end{array}$$ (1)

where *s*(*i*) is the silhouette coefficient of data point *i*, *a*(*i*) is the average distance between *i* and all the other data points in the cluster to which *i* belongs, *a*(*i*) represents the intra-cluster dissimilarity of sample *i*, *b*(*i*) is the minimum average distance from *i* to all clusters to which *i* does not belong. The inter-cluster dissimilarity of sample *i* is defined as *b*(*i*).

### R Shiny

The Shiny package for R [35] was used to develop the web application since the data were analysed also using R statistical software [32].

Shiny is a R web application framework that allows the development of interactive web applications. This package makes it easy to create websites that interact with R without prior knowledge of web programming or other scripting languages. To create the Shiny application, we uploaded the dataset, built the clustering algorithm in R, and then used these two files to create the Shiny application. A brief summary of Shiny and a description of the main components used to implement the application are provided in S2 Appendix. The program allows user involvement and generates interactive visualisations, such as maps with padding and zooming capabilities. One disadvantage of Shiny is that applications created with it can only be deployed online using the Shiny web server. It is noted, however, that although Shiny currently has a relatively limited feature set, this will likely expand, given the product’s popularity [49].

It is important to note that the application requires access to the internet.

## Results

**Fig 1:**
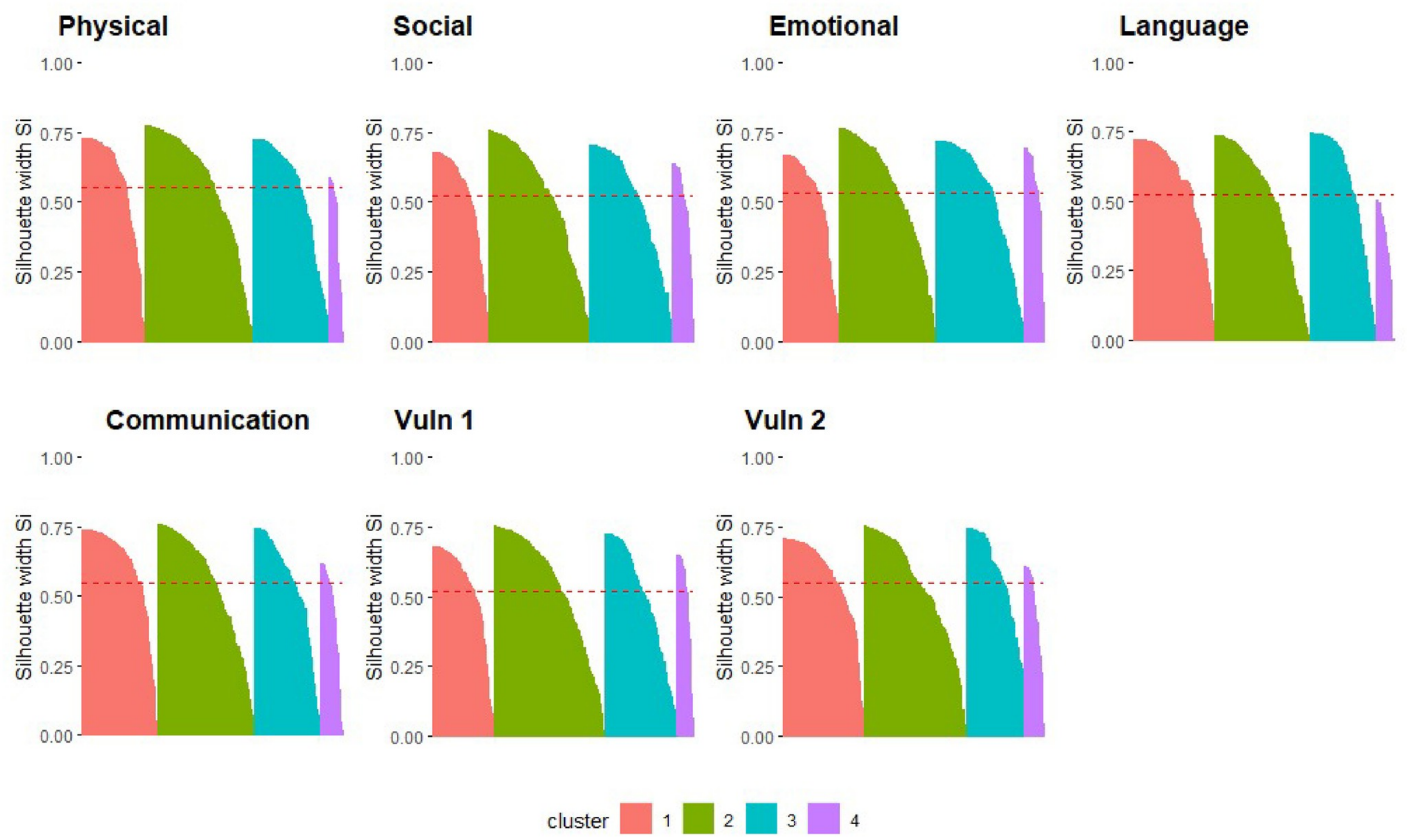
Silhouette plots for clusters across the five AEDC domains and two indicators, the *x*-axis are the clusters, and the height of each cluster is the silhouette width score for the cluster. The dotted line is the average silhouette width score across the four clusters.

The developmentally vulnerable proportions were analysed on the log scale, due to skewness in the proportions, and converted back to their original scale in reporting the results. The number of clusters was evaluated for *K* = {3, 4, 5, …, 12}, separately for each of the five AEDC domains and the two composite domain indicators (Vuln 1, Vuln 2). The optimal number of clusters for each domain was chosen to be four after validating the clusters internally using silhouette scores. The silhouette plots for the clusters in the five domains and two indicators are displayed in Fig 1.

Summary statistics for each cluster (size, mean, variance, range) for the five AEDC domains and two indicators with the associated demographic factors are given in S1 Appendix. These results are visualised in the R Shiny application, accessed at https://waladraidi.shinyapps.io/Shiny_2_6_2022/.

**Fig 2:**
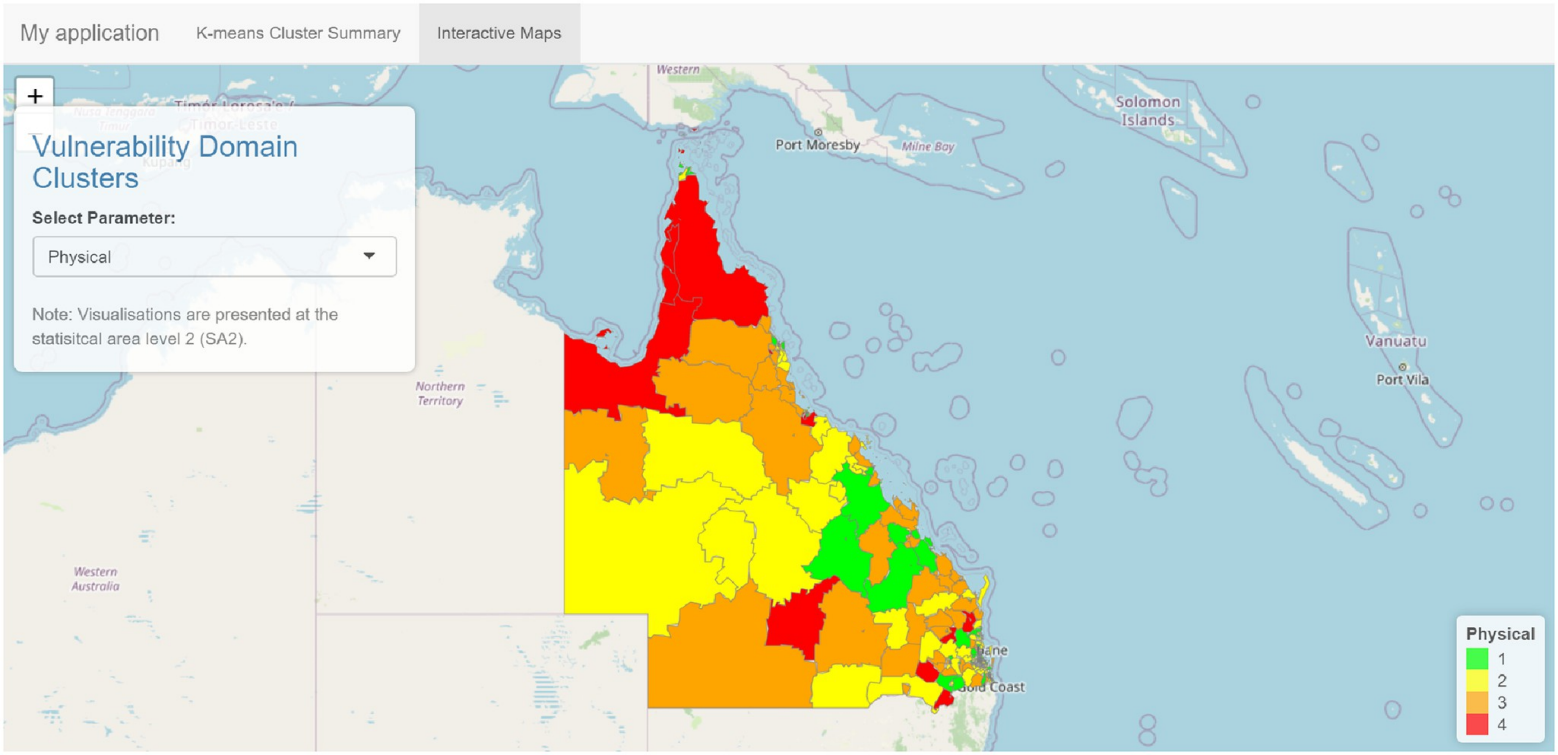
Example of *K*-means clustering results displayed in the web interface for C1 of the physical health development domain; the dashboard shows the box plot for the proportions of Australia, English, Indigenous and Preschool variables, and pie charts for the percentages of remoteness and IRSD and the location of C1 on a Queensland map.

**Fig 3:**
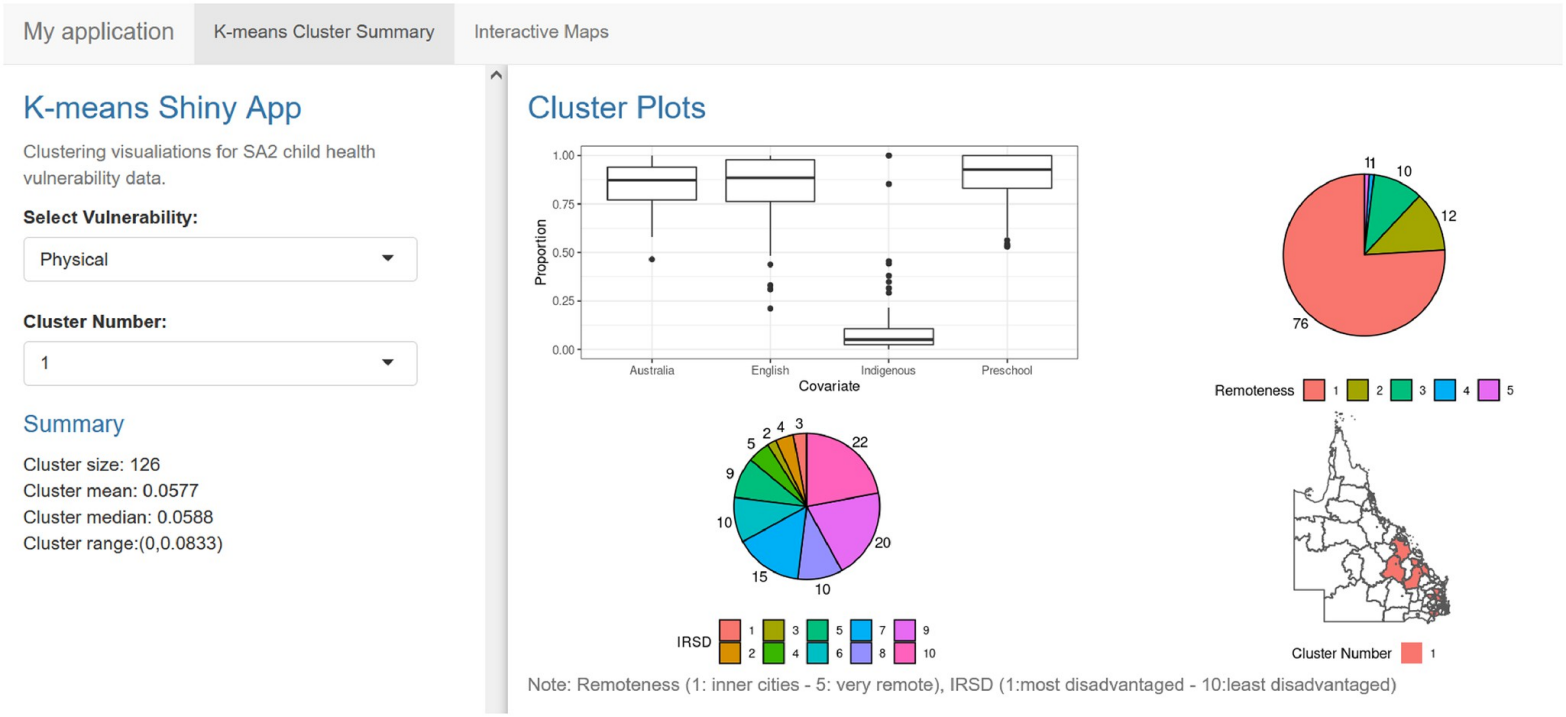
Map of the four clusters obtained for the physical health AEDC domain. The clusters are ordered from C1 (green, least vulnerable) to C4 (red, most vulnerable).

Figs 2 and 3 illustrate the application interface. The interface includes two tabs. For the first tab (Fig 2), which shows the *K*-means cluster summary, the user can select the type of development vulnerability and the cluster of interest. The clusters, labelled C1, C2, C3 and C4, correspond to vulnerability levels ordered from lowest vulnerability (C1) to highest vulnerability (C4). Furthermore, this first tab shows the associated characteristics related to the demographic factors for each cluster and the location of the SA2 areas on the map. The second tab (Fig 3) shows a map of the distribution of the clusters (regions of differing vulnerability) for a given development vulnerability. The user can choose the type of AEDC domain from the five domains and two indicators and can zoom in on the Queensland map to view finer details for each region. This provides an interactive visual summary of vulnerability across the regions of Queensland and a comparison of the vulnerabilities across the five AEDC domains and two indicators. An example map is given in Fig 3. A selection of outputs from the app, comparing C4 (highest vulnerability) to C1 (lowest vulnerability), is provided in S2 Appendix.

In comparing the most vulnerable cluster, C4, to the least vulnerable cluster, C1, there are higher proportions of children who do not have English as their mother language, who are Indigenous and who did not attend preschool (Table 1, and Table 8 from S1 Appendix). The one exception to this is that, for the SA2s making up the most emotionally vulnerable cluster, C4, there is a higher proportion of children who do have English as their mother language compared to the least emotionally vulnerable cluster, C1 (Table 8 from S1 Appendix). The SA2s belonging to C4 are located in far-north and north-west Queensland, and a small number can be found in the coastal areas of Queensland (Table 9 from S1 Appendix). In contrast, the SA2s belonging to C1 can be found in the south-east of the state. This region contains the majority of the children of Queensland and the capital city, Brisbane (Table 9 from S1 Appendix). Across all AEDC domains, there is a much higher proportion of children residing in SA2s belonging to C4 with a low IRSD score (greater socio-economic disadvantage) compared to C1.

**Table 1:** Comparison of clusters, C4 (most vulnerable) and C1 (least vulnerable), averaged over the five AEDC domains (excluding Vuln 1 and Vuln 2). The percentage of children where “English Not Primary Language” was calculated as 1 minus “English”. Similarly, “No Preschool” is 1 minus “Preschool”.

	C4(%)		C1(%)	
	mean	sd	mean	sd
English Not Primary Language	19.0	12.7	14.4	1.50
Indigenous	39.6	17.1	9.20	1.60
No Preschool	23.2	1.80	11.8	0.80
Remoteness – Cities	31.8	10.6	70.2	5.90
Remoteness – Regional	49.2	6.40	28.0	5.10
Remoteness – Remote	11.4	5.60	2.40	0.90
IRSD – Low	84.4	13.2	16.2	6.20

In comparing Vuln 1 and Vuln 2, unsurprisingly, the proportion of children who are vulnerable on two or more (2+) domains is lower than the proportion vulnerable on one or more (1+) domains for both C4 and C1. For C4, the proportion of children who don’t have English as their primary language is higher for 2+ vulnerabilities compared to 1+ vulnerabilities. The proportion of children who identified as Indigenous is lower for 2+ vulnerabilities compared to 1+ vulnerabilities. The proportion who did not attend preschool is about the same for 1+ and 2+ vulnerabilities. The SA2s belonging to C4 for Vuln 1 are located in the same geographic areas of Queensland as Vuln 2 and additionally in the south-east and central coast. For the SA2s in C4, there is a higher proportion of children residing in SA2s with a low IRSD score (greater socio-economic disadvantage) for Vuln 1 compared to Vuln 2.

In comparison, across the five domains for the most vulnerable cluster (C4), the smallest cluster size can be found in the physical health domain with around 30 SA2 areas, and the largest cluster size can be found in the communication skills domain, 46 SA2 areas.

The variation in cluster sizes between the physical health domain and the communication skills domain in the most vulnerable cluster (C4) could be attributed to multiple factors. Firstly, the larger cluster size for communication might indicate a broader, systemic issue affecting a larger geographic area or demographic. Secondly, challenges in the communication domain might be easier to identify or measure compared to physical health. For example, communication difficulties can manifest in daily interactions and academic settings, leading to easier identification, whereas certain physical health challenges might be latent or less obvious without comprehensive medical assessments [50]. Thirdly, the regions and demographics within the communication skills domain cluster might share unique socio-cultural attributes that impact communication skills development. It could be influenced by factors like parental education, exposure to multiple languages, or limited access to early childhood education.

In the physical domain, we observed a notably higher proportion of Indigenous children, potentially due to a combination of factors. Historical challenges, barriers to healthcare access, unique cultural practices, environmental conditions, and socio-economic disparities can impact Indigenous children’s physical health [51]. This was especially prominent in areas like Northern Queensland. While the country of birth exceeded 85% consistently across all domains and clusters, variations between clusters remained minimal at around 5%. The elevated representation of Indigenous children in the Physical domain suggests unique regional challenges or attributes in places with significant Indigenous populations, necessitating specialized interventions (see S3 Appendix).

## Discussion

This study is grounded in addressing specific gaps in the literature related to children’s development and vulnerability in Queensland, Australia. Firstly, one of the primary contributions of our study is the comprehensive analysis of children’s vulnerability across multiple domains. Unlike many previous studies that often focus on specific aspects of children’s development, our research conducts cluster analysis for each of the five AEDC domains and two indicators. This approach provides a more holistic understanding of vulnerability, recognizing that it can manifest differently across various developmental dimensions. Secondly, our study introduces a spatial dimension to the analysis. By clustering SA2 areas based on vulnerability, we identify regions with high vulnerabilities. This spatial aspect adds a layer of understanding that is not achievable through SEIFA data alone. It helps policymakers and health managers target interventions to specific geographic areas where vulnerable children are more prevalent. Thirdly, we developed an interactive R Shiny application to enhance accessibility and usability, empowering stakeholders like health managers and policymakers to explore regions of high vulnerability beyond the limitations of SEIFA data, offering a dynamic platform for interaction. Lastly, our study provides practical implications for improving children’s development outcomes. We highlight the need for targeted interventions in highly vulnerable regions, enhanced early children’s education programs, socioeconomic support to reduce disparities, and culturally sensitive services for Indigenous communities. These recommendations are based on the specific vulnerabilities identified through clustering.

Our primary objective in this study is to investigate the clustering of vulnerable children in different geographical regions within Queensland, Australia. However, it is essential to acknowledge that children’s vulnerability is a multifaceted phenomenon influenced by various individual and contextual factors. In some instances, we have discussed these other characteristics to provide a more comprehensive context for the interpretation of cluster patterns within the designated geographical regions. The mention of characteristics such as the proportion of Indigenous children or the country of birth within the clusters is intended to offer insights into the diversity of vulnerable children across regions. While the geographical location is the central focus of our analysis, understanding the demographic and socio-economic composition of these regions is essential for policymakers and stakeholders when developing targeted interventions. Our emphasis on geography stems from the uniqueness of this paper in investigating clustering patterns in children’s vulnerability within specific geographic areas. We intend to make this connection clearer in the paper, emphasizing how these characteristics intersect with geography to provide a holistic view of children’s vulnerability in Queensland’s different regions. This approach aligns with the paper’s overarching goal of informing targeted interventions and policies to support vulnerable children in geographically distinct areas. Our primary motivation for clustering each AEDC domain separately was the hypothesis that each domain might exhibit unique geographical patterns. For instance, areas with resources that support physical activities might have lower vulnerability in the physical domain but may not necessarily influence the emotional or social domains in the same way. Additionally, by studying the clusters for each domain individually, we aimed to identify if certain geographical areas consistently showed up as vulnerable across multiple domains. This would allow us to pinpoint areas that require multifaceted interventions. We acknowledge the concern about the potential overload of information with 28 clusters. Given the value of examining individual AEDC domains, we discussed the findings from the ‘Vuln1’ and ‘Vuln2’ clusters these provide a comprehensive overview of vulnerability, and for the individual AEDC domains, we provide a summarized, higher-level overview, focusing on the most striking and relevant patterns, rather than delving deep into each cluster and provide the deep comparison in S3 Appendix.

The current study employs cluster analysis to comprehensively understand children’s development in various domains, aligning with Brinkman’s findings [10] on the association between low socioeconomic status and developmental vulnerabilities. In addition to previous research utilizing clustering techniques, our study introduces a spatial dimension, revealing vulnerability clusters in Queensland. This spatial insight adds a crucial layer to the existing literature, helping identify regions with concentrated vulnerabilities. The use of K-means clustering facilitates region-based analysis and policy development, aiding stakeholders, including health managers and policymakers, in addressing children’s vulnerabilities in different areas.

Demographic differences between C4 and C1 clusters are significant. C4 has more children with English as a secondary language, Indigenous children, and those not attending preschool. In C4’s emotionally vulnerable cluster, more children have English as their primary language compared to C1. C4 is concentrated in far north and northwest Queensland, while C1 is primarily in the southeast, including Brisbane. For both Vuln 1 and Vuln 2 indicators, C4 has lower proportions of children vulnerable in one domain (1+), but higher proportions for those vulnerable in two or more domains (2+), especially with non-English primary languages. C4 cluster sizes are larger, with higher percentages of inner-city, regional, and very remote areas. C4 consistently has more socioeconomically disadvantaged and Indigenous children. Geographically, C4 is concentrated in the far north, highlighting domain-specific vulnerability linked to socio-economic factors.

The study highlights the link between specific geographic locations in Queensland and distinct clusters of vulnerabilities among children. The northern parts of Queensland, extending to the central coast and southeast regions, are significant core areas for physical health vulnerabilities. This is obvious from the marked percentage of children in very remote areas and those from socioeconomically disadvantaged backgrounds. Similarly, the northwest region, especially in and around central Queensland, is the centre for social vulnerabilities, further increased by the number of children from very remote locations. The far north of Queensland consistently emerges as a critical region of concern, especially for emotional and language vulnerabilities, underlined by the high percentages of children from remote areas, lower English-first language speakers, and a significant Indigenous population. Communication vulnerabilities have a strong footprint in the northwest, with evidence also present in the southwest and coastal regions.

The study discussed the variation in cluster sizes across the AEDC domains, the communication skills domain (Communication) was found to have the largest cluster size for the most vulnerable SA2s (C4) compared to the other domains. In contrast, the language and cognitive skill domain (Language) had the largest cluster size for the least vulnerable SA2s (C1) compared to other domains, one possible explanation for the larger cluster size in the Communication domain among vulnerable SA2s (C4) could be the higher prevalence of communication challenges among children. This domain encompasses a wide range of skills, including speech articulation, and social communication [52]. Further research could explore whether certain risk factors, such as limited access to speech therapy or exposure to adverse environmental conditions, are more widespread in specific regions, thus affecting communication skills across a broader population. Some factors contributing to communication difficulties, such as limited access to educational resources or early intervention services, may be more geographically dispersed, affecting children in a wider range of areas [53]. In contrast, the factors contributing to vulnerability in the Language and Cognitive Skills domain may be more concentrated in specific regions, leading to the observed cluster patterns. Particularly in remote areas, may contribute to communication difficulties among children [54]. Regions with a higher percentage of children with diverse linguistic backgrounds may face unique challenges in language development, which could be reflected in the cluster patterns. It is crucial to note that the analysis was conducted at the SA2 level of aggregation, and care must be applied when making inferences at different levels or about individual children due to potential biases, such as Simpson’s paradox.

This paper extends and aligns with previous research by conducting a comprehensive cluster analysis across multiple domains of children’s development, considering the spatial aspect of vulnerability, and developing an interactive visualization tool. It contributes valuable insights to the existing body of knowledge and offers practical implications for improving children’s development outcomes in Queensland, Australia. The findings highlight the critical need for targeted interventions in regions with high vulnerability, especially those falling under the C4 clusters. Policymakers and health managers should prioritize these areas to ensure that vulnerable children receive the necessary support and resources. Additionally, addressing the strong link between vulnerability and socioeconomic disadvantage is essential. Social support programs and policies should focus on reducing disparities in economic well-being among families with vulnerable children. Furthermore, the lower proportion of preschool attendance among vulnerable clusters suggests a need for enhanced early childhood education programs. Investment in accessible and quality preschool education can play a pivotal role in improving children’s development outcomes. Given the higher proportions of Indigenous children in vulnerable clusters, interventions and services should be culturally sensitive and designed to meet the unique needs of Indigenous communities. Policymakers and stakeholders should adopt a holistic approach to children’s development, recognizing that vulnerability can manifest across various domains. Regular assessments will help determine the effectiveness of policies and programs in reducing children’s vulnerability and guide adjustments as needed.

## Supporting information

S1 Appendix Results from *K*-means algorithm for each type of Children developmental vulnerability. For all results, the clusters are ordered from lowest vulnerability (C1) to highest vulnerability (C4).
(PDF)

S2 Appendix R Shiny. (PDF)

S3 Appendix Results from each type of AEDC domain. (PDF)
