# Supplementary material for: Where are the vulnerable children? Identification and comparison of clusters of young children with health and developmental vulnerabilities across Queensland
Source: PLoS One. 2024 Mar 15;19(3):e0298532. doi: 10.1371/journal.pone.0298532 (PMC10942074; doi:10.1371/journal.pone.0298532)
Supplement: S1 Appendix — For all results, the clusters are ordered from lowest vulnerability (C1) to highest vulnerability (C4). (PDF) [file pone.0298532.s001.pdf]

S1 Appendix. Results from K-means algorithm for each type of developmentally vulnerable. For all results, the clusters are ordered from lowest vulnerability (C1) to highest vulnerability (C4).

Table 1: K-means results for physical health domain vulnerability (Physical), where n is the number of SA2's in the cluster.

	C1 (n=126)		C2 (n=219)		C3 (n=151)		C4 (n=30)	
	mean	range	mean	range	mean	range	mean	range
**Domain**								
Physical	0.06	(0.00, 0.08)	0.11	(0.08, 0.14)	0.17	(0.14, 0.22)	0.28	(0.22, 0.68)
**Demographic**								
Australia	0.85	(0.46, 1.00)	0.88	(0.41, 1.00)	0.89	(0.52, 1.00)	0.89	(0.72, 1.00)
English	0.85	(0.21, 1.00)	0.81	(0.06, 1.00)	0.83	(0.34, 1.00)	0.69	(0.01, 1.00)
Indigenous	0.10	(0.00, 1.00)	0.13	(0.00, 1.00)	0.21	(0.00, 0.83)	0.40	(0.00, 1.00)
Preschool	0.89	(0.53, 1.00)	0.85	(0.44, 1.00)	0.78	(0.44, 1.00)	0.7579	(0.46, 1.00)
Remoteness								
Inner cities	0.76		0.56		0.43		0.23	
Inner regional	0.12		0.22		0.28		0.27	
Outer regional	0.10		0.18		0.24		0.30	
Remote	0.01		0.02		0.03		0.03	
Very remote	0.01		0.02		0.02		0.17	
IRSD								
1 (low)	0.03		0.05		0.15		0.50	
2	0.04		0.07		0.18		0.14	
3	0.02		0.08		0.16		0.30	
4	0.05		0.12		0.12		0.00	
5	0.09		0.11		0.10		0.00	
6	0.10		0.17		0.11		0.00	
7	0.15		0.10		0.05		0.03	
8	0.10		0.13		0.07		0.00	
9	0.20		0.09		0.03		0.03	
10 (high)	0.22		0.08		0.03		0.00	

Table 2: K-means results for social competence domain vulnerability (Social), where n is the number of SA2's in the cluster.

	C1 (n=111)		C2 (n=205)		C3 (n=168)		C4 (n=42)	
	mean	range	mean	range	mean	range	mean	range
**Domain**								
Social	0.04	(0.00, 0.07)	0.097	(0.07, 0.12)	0.15	(0.12, 0.19)	0.23	(0.19, 0.39)
**Demographic**								
Australia	0.85	(0.41, 1.00)	0.88	(0.53, 1.00)	0.89	(0.46, 1.00)	0.89	(0.53, 1.00)
English	0.84	(0.14, 1.00)	0.82	(0.21, 1.00)	0.82	(0.19, 1.00)	0.75	(0.01, 1.00)
Indigenous	0.10	(0.00, 0.85)	0.15	(0.00, 1.00)	0.19	(0.00, 1.00)	0.28	(0.00, 1.00)
Preschool	0.88	(0.44, 1.00)	0.84	(0.45, 1.00)	0.81	(0.44, 1.00)	0.78	(0.46, 1.00)
Remoteness								
Inner cities	0.66		0.57		0.51		0.45	
Inner regional	0.20		0.19		0.26		0.19	
Outer regional	0.11		0.20		0.18		0.29	
Remote	0.02		0.01		0.03		0.00	
Very remote	0.01		0.03		0.02		0.07	
IRSD								
1 (low)	0.02		0.07		0.13		0.31	
2	0.04		0.05		0.16		0.24	
3	0.04		0.09		0.14		0.12	
4	0.08		0.13		0.08		0.04	
5	0.09		0.12		0.08		0.05	
6	0.09		0.14		0.15		0.07	
7	0.14		0.09		0.08		0.05	
8	0.11		0.12		0.08		0.05	
9	0.21		0.10		0.04		0.02	
10 (high)	0.18		0.09		0.06		0.05	

Table 3: K-means results for emotional maturity domain vulnerability (Emotional), where n is the number of SA2's in the cluster.

	C1 (n=113)		C2 (n=194)		C3 (n=180)		C4 (n=39)	
	mean	range	mean	range	mean	range	mean	range
**Domain**								
Emotional	0.05	(0.00, 0.07)	0.09	(0.07, 0.10)	0.13	(0.11, 0.16)	0.20	(0.17, 0.27)
**Demographic**								
Australia	0.87	(0.41, 1.00)	0.87	(0.46, 1.00)	0.89	(0.53, 1.00)	0.9034	(0.70, 1.00)
English	0.86	(0.21, 1.00)	0.82	(0.27, 1.00)	0.81	(0.14, 1.00)	0.77	(0.01, 1.00)
Indigenous	0.11	(0.00, 0.85)	0.14	(0.00, 1.00)	0.19	(0.00, 1.00)	0.28	(0.00, 1.00)
Preschool	0.87	(0.44, 1.00)	0.84	(0.45, 1.00)	0.82	(0.44, 1.00)	0.78	(0.45, 0.99)
Remoteness								
Inner cities	0.64		0.56		0.51		0.51	
Inner regional	0.18		0.22		0.24		0.20	
Outer regional	0.15		0.19		0.19		0.21	
Remote	0.02		0.01		0.03		0.00	
Very remote	0.01		0.02		0.03		0.08	
IRSD								
1 (low)	0.03		0.05		0.13		0.38	
2	0.02		0.08		0.17		0.10	
3	0.07		0.08		0.12		0.18	
4	0.14		0.10		0.07		0.05	
5	0.10		0.09		0.13		0.00	
6	0.10		0.16		0.12		0.08	
7	0.09		0.13		0.06		0.10	
8	0.11		0.12		0.08		0.03	
9	0.16		0.10		0.06		0.05	
10 (high)	0.18		0.09		0.06		0.03	

Table 4: K-means results for language domain vulnerability (Language), where n is the number of SA2's in the cluster.

	C1 (n=162)		C2 (n=194)		C3 (n=133)		C4 (n=37)	
	mean	range	mean	range	mean	range	mean	range
**Domain**								
Language	0.03	(0.00, 0.05)	0.08	(0.06, 0.11)	0.14	(0.11, 0.21)	0.29	(0.22, 0.55)
**Demographic**								
Australia	0.85	(0.46, 1.00)	0.89	(0.41, 1.00)	0.90	(0.59, 1.00)	0.89	(0.73, 1.00)
English	0.85	(0.14, 1.00)	0.82	(0.21, 1.00)	0.79	(0.01, 1.00)	0.56	(0.06, 0.83)
Indigenous	0.07	(0.00, 0.64)	0.16	(0.00, 0.85)	0.27	(0.00, 1.00)	0.69	(0.15, 1.00)
Preschool	0.89	(0.45, 1.00)	0.82	(0.44, 1.00)	0.76	(0.45, 1.00)	0.78	(0.46, 0.97)
Remoteness								
Inner cities	0.77		0.52		0.32		0.00	
Inner regional	0.12		0.26		0.29		0.18	
Outer regional	0.11		0.19		0.27		0.36	
Remote	0.00		0.03		0.03		0.09	
Very remote	0.00		0.00		0.08		0.36	
IRSD								
1 (low)	0.01		0.07		0.21		0.82	
2	0.02		0.09		0.23		0.09	
3	0.02		0.11		0.21		0.09	
4	0.05		0.12		0.13		0.00	
5	0.08		0.13		0.08		0.00	
6	0.11		0.19		0.07		0.00	
7	0.15		0.10		0.01		0.00	
8	0.13		0.11		0.03		0.00	
9	0.19		0.06		0.02		0.00	
10 (high)	0.22		0.03		0.01		0.00	

Table 5: K-means results for communication skills domain vulnerability (Communication), where n is the number of SA2's in the cluster.

	C1 (n=152)		C2 (n=195)		C3 (n=133)		C4 (n=46)	
	mean	range	mean	range	mean	range	mean	range
**Domain**								
Communication	0.04	(0.00, 0.07)	0.09	(0.07, 0.12)	0.15	(0.12, 0.18)	0.22	(0.18, 0.43)
**Demographic**								
Australia	0.88	(0.41, 1.00)	0.86	(0.59, 1.00)	0.89	(0.63, 1.00)	0.88	(0.67, 1.00)
English	0.88	(0.58, 1.00)	0.84	(0.21, 1.00)	0.77	(0.14, 1.00)	0.65	(0.01, 1.00)
Indigenous	0.08	(0.00, 0.46)	0.14	(0.00, 0.85)	0.24	(0.00, 1.00)	0.33	(0.00, 1.00)
Preschool	0.88	(0.44, 1.00)	0.85	(0.46, 1.00)	0.78	(0.45, 1.00)	0.74	(0.44, 1.00)
Remoteness								
Inner cities	0.68		0.58		0.43		0.40	
Inner regional	0.18		0.21		0.26		0.19	
Outer regional	0.13		0.18		0.21		0.27	
Remote	0.01		0.02		0.03		0.03	
Very remote	0.00		0.01		0.07		0.11	
IRSD								
1 (low)	0.01		0.06		0.17		0.38	
2	0.03		0.07		0.19		0.24	
3	0.03		0.08		0.19		0.13	
4	0.06		0.11		0.12		0.11	
5	0.07		0.12		0.12		0.02	
6	0.15		0.17		0.07		0.03	
7	0.12		0.13		0.05		0.00	
8	0.15		0.09		0.06		0.03	
9	0.16		0.11		0.01		0.03	
10 (high)	0.22		0.06		0.02		0.03	

Table 6: K-means results for vulnerability on one or more domain(s) (Vuln 1), where n is the number of SA2's in the cluster.

	C1 (n=101)		C2 (n=181)		C3 (n=173)		C4 (n=71)	
	mean	range	mean	range	mean	range	mean	range
**Domain**								
Vuln 1	0.15	(0.06, 0.19)	0.24	(0.19, 0.28)	0.29	(0.28, 0.38)	0.40	(0.38, 0.71)
**Demographic**								
Australia	0.85	(0.46, 1.00)	0.87	(0.41, 1.00)	0.89	(0.53, 1.00)	0.90	(0.66, 1.00)
English	0.88	(0.46, 1.00)	0.84	(0.29, 1.00)	0.81	(0.14, 1.00)	0.6993	(0.01, 1.00)
Indigenous	0.07	(0.00, 0.38)	0.13	(0.00, 1.00)	0.19	(0.00, 0.93)	0.33	(0.00, 1.00)
Preschool	0.90	(0.44, 1.00)	0.85	(0.54, 1.00)	0.80	(0.44, 1.00)	0.79	(0.45, 1.00)
Remoteness								
Inner cities	0.75		0.56		0.46		0.35	
Inner regional	0.15		0.22		0.26		0.23	
Outer regional	0.09		0.18		0.22		0.21	
Remote	0.01		0.02		0.03		0.03	
Very remote	0.00		0.02		0.03		0.18	
IRSD								
1 (low)	0.00		0.03		0.14		0.50	
2	0.02		0.04		0.22		0.12	
3	0.05		0.06		0.17		0.26	
4	0.06		0.11		0.10		0.09	
5	0.08		0.13		0.12		0.00	
6	0.11		0.13		0.11		0.03	
7	0.10		0.17		0.05		0.00	
8	0.14		0.16		0.03		0.00	
9	0.20		0.11		0.02		0.00	
10 (high)	0.24		0.10		0.04		0.00	

Table 7: K-means results for vulnerability on two or more domains (Vuln 2), where n is the number of SA2's in the cluster.

	C1 (n=162)		C2 (n=207)		C3 (n=117)		C4 (n=40)	
	mean	range	mean	range	mean	range	mean	range
**Domain**								
Vuln 2	0.07	(0.00, 0.10)	0.13	(0.10, 0.16)	0.19	(0.16, 0.23)	0.28	(0.24, 0.55)
**Demographic**								
Australia	0.86	(0.46, 1.00)	0.88	(0.41, 1.00)	0.89	(0.53, 1.00)	0.90	(0.72, 1.00)
English	0.85	(0.14, 1.00)	0.83	(0.14, 1.00)	0.81	(0.31, 100)	0.66	(0.01, 1.00)
Indigenous	0.13	(0.00, 1.00)	0.33	(0.00, 1.00)	0.07	(0.00, 0.38)	0.19	(0.00, 0.93)
Preschool	0.85	(0.54, 1.00)	0.79	(0.45, 1.00)	0.90	(0.44, 1.00)	0.80	(0.44, 1.00)
Remoteness								
Inner cities	0.70		0.57		0.42		0.35	
Inner regional	0.17		0.20		0.29		0.25	
Outer regional	0.13		0.18		0.22		0.25	
Remote	0.00		0.02		0.03		0.02	
Very remote	0.00		0.03		0.04		0.13	
IRSD								
1 (low)	0.02		0.04		0.15		0.52	
2	0.03		0.06		0.22		0.20	
3	0.04		0.07		0.19		0.20	
4	0.07		0.13		0.10		0.02	
5	0.09		0.12		0.11		0.00	
6	0.10		0.20		0.08		0.0	
7	0.13		0.12		0.04		0.03	
8	0.12		0.13		0.05		0.00	
9	0.20		0.07		0.02		0.03	
10 (high)	0.20		0.06		0.04		0.00	

Table 8: Comparison of C4 (most vulnerable) to C1 (least vulnerable) for the five domains of development and two indicators.

	Physical		Social		Emotional		Language		Communication		Vuln 1		Vuln 2	
Cluster size	C4(%) (n-30)	C1(%) (n=126)	C4(%) (n=42)	C1(%) (n=111)	C4(%) (n=39)	C1(%) (n=113)	C4(%) (n=37)	C1(%) (n=162)	C4(%) (n=46)	C1(%) (n=152)	C4(%) (n=71)	C1(%) (n=101)	C4(%) (n=40)	C1(%) (n=162)
Vulnerable	28	6	24	5	20	5	29	3	22	4	40	15	28	7
English not primary language	31	15	25	16	10	14	44	15	35	12	30	12	34	15
Indigenous	40	10	28	10	28	11	69	7	33	8	33	7	19	13
No preschool	24	11	22	12	22	13	22	11	26	12	21	10	20	15
Remoteness – Cities	23	76	45	66	51	64	0	77	40	68	35	75	35	70
Remoteness – Regional	57	22	48	31	41	33	54	23	46	31	44	24	50	30
Remoteness – Remote	20	2	7	3	8	3	8	3	14	1	21	1	15	0
IRSD – Low	94	14	71	18	71	26	100	10	86	13	97	13	94	16

Table 9: Geographic locations of the most vulnerable cluster (C4) and the least vulnerable cluster (C1).

	Physical	Social	Emotional	Language	Communication	Vuln 1	Vuln 2
C4	Far North, very small number Central Queensland, small number South-East	North-west, small number Central Queensland	North-west, small number in Central Queensland	Far north	North-west, small number in south west and coastal areas	Far North, very small number Central coast, small number south east	Far North, small number south east
C1	South east and Central Queensland	South-East and part of Central Queensland few regions in the Central west	North-west, small number in Central Queensland	South-East and coastal area	South-East and Central Queensland, few regions in Cairns	South-East and Central Queensland	South-East and Central Queensland
