# Supplementary material for: Where are the vulnerable children? Identification and comparison of clusters of young children with health and developmental vulnerabilities across Queensland
Source: PLoS One. 2024 Mar 15;19(3):e0298532. doi: 10.1371/journal.pone.0298532 (PMC10942074; doi:10.1371/journal.pone.0298532)
Supplement: S2 Appendix — (PDF) [file pone.0298532.s002.pdf]

S2 Appendix. R shiny

The following description is based on the explanation given in Moraga's book [2], Chapter thirteen, pages 203 - 215.
R shiny is a web interactive interface used to build applications in the statistical software package R. Two R scripts are required: a user-interface script called `ui.R` and a server script called `server.R`. The user interface script is in control of the application's layout and appearance. The server script contains the R objects as well as the instructions for displaying them. Shiny applications support interactivity by utilising a feature known as reactivity. Users can enter text, select dates, or change other inputs in this manner, and the R objects displayed will change automatically.
The steps below can be used to create reactive objects. Reactive expressions let you control which parts of the application (app) update and prevent unnecessary computation that can slow down the app). R objects are first added to the user interface. This is accomplished by including output functions in the `ui.R` script that converts R objects to output. Following that, the R code for creating the objects is provided in the `server.R`. This script includes an unnamed function as well as two list-like objects called output and input. Input stores the current values of the objects in the application, while output contains all of the instructions for building the R objects. The objects are created with a render function and saved in the output list. By including an input value in a `render*` expression, reactivity is created.
To make a reactive plot, for example, we need to include a `plotOutput` function in the `ui.R`. The plot is then created using a `renderPlot` function and added to the output object `server.R` [1].
The following graphs, generated from the Shiny app, display the profiles of the covariates for each domain in categories C1 and C4.

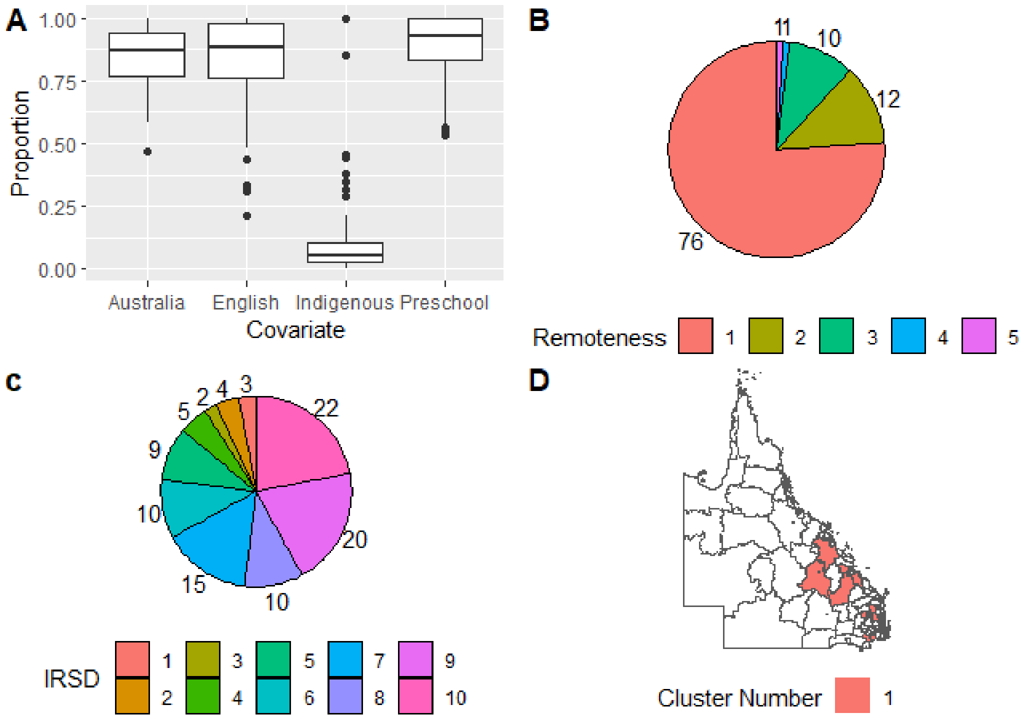

Figure 1: First cluster for Physical using K-means, SA2's size=126 (lowest SA2 proportion of Physical vulnerability).

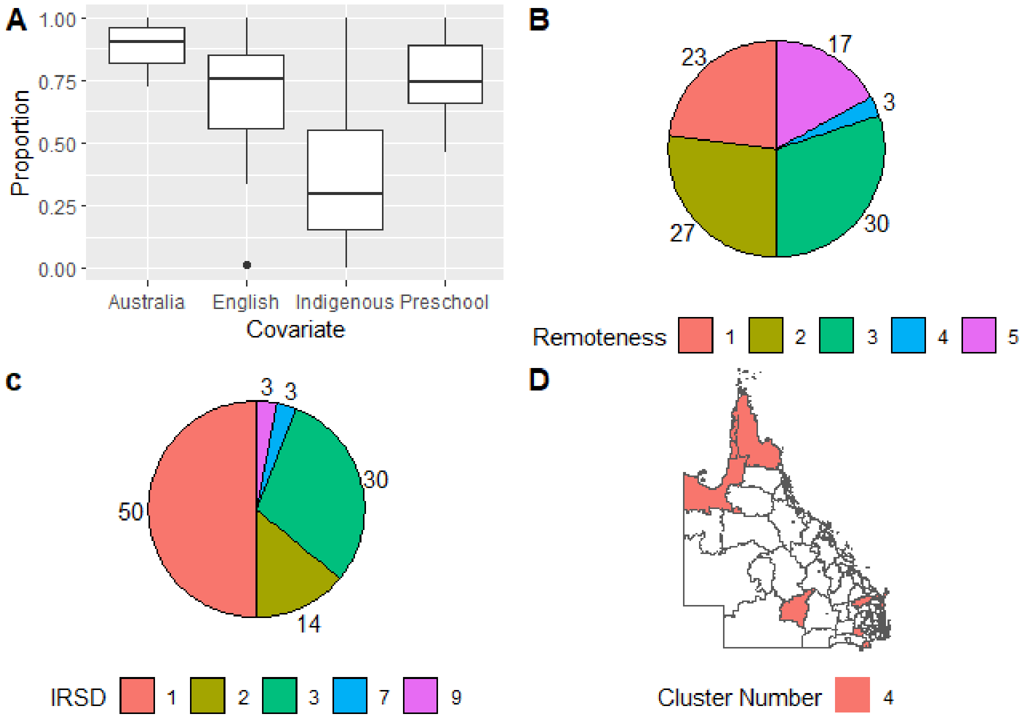

Figure 2: Fourth cluster for Physical using K-means, SA2's size=30 (highest SA2 proportion of Physical vulnerability).

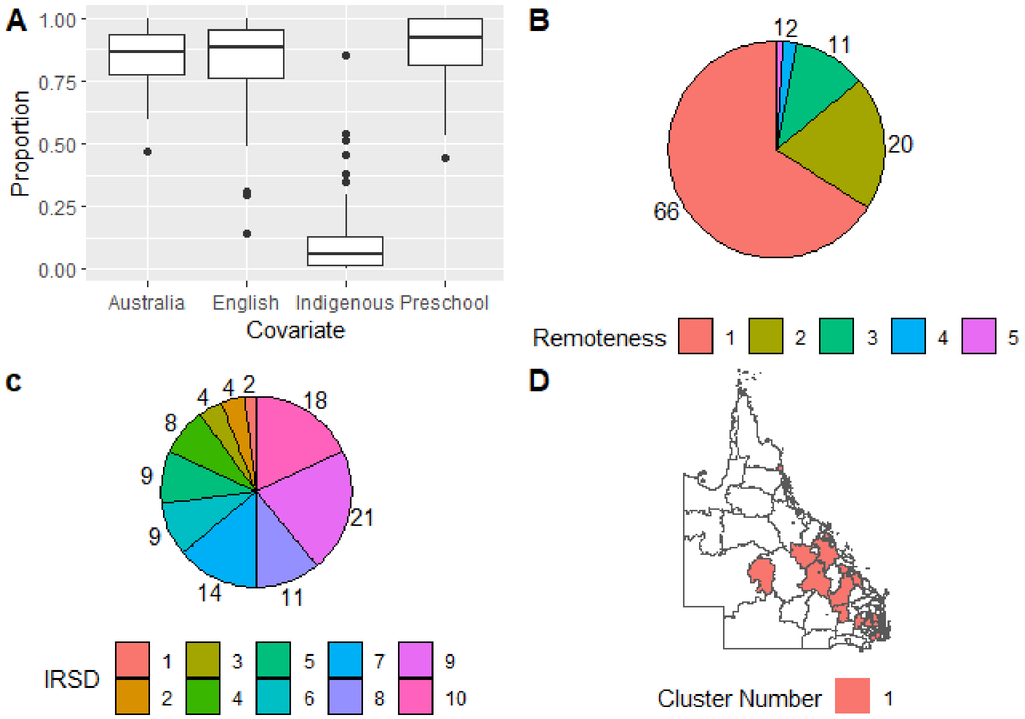

Figure 3: First cluster for Social using K-means, SA2's size=111 (lowest SA2 proportion of Social vulnerability).

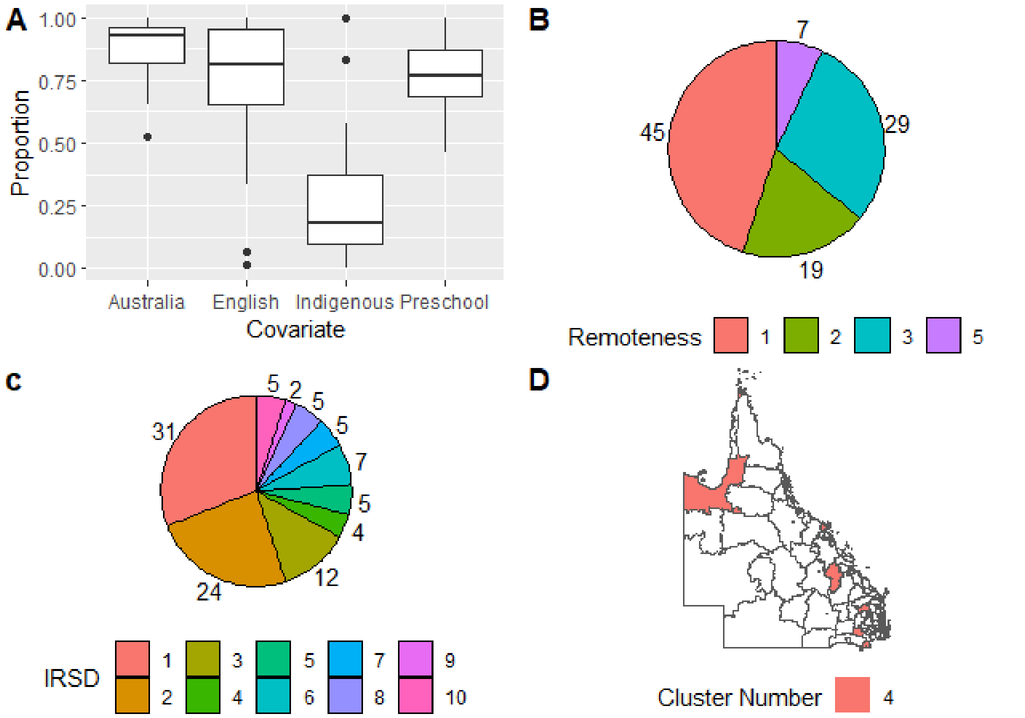

Figure 4: Fourth cluster for Social using K-means, SA2's size=42 (highest SA2 proportion of Social vulnerability).

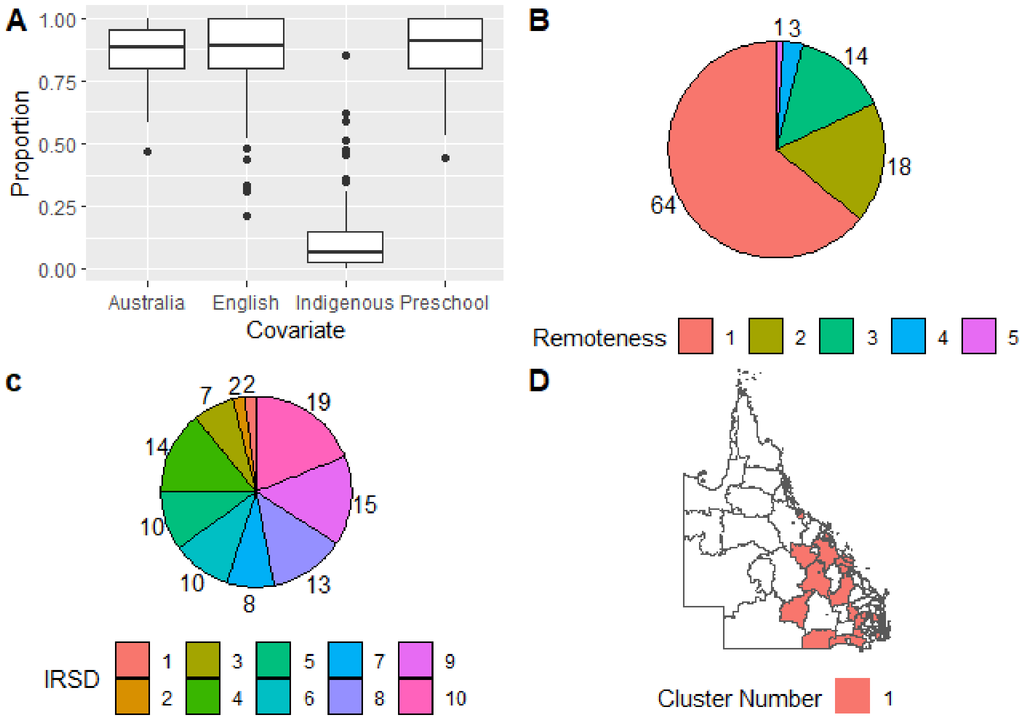

Figure 5: First cluster for Emotional using K-means, SA2's size=113 (lowest SA2 proportion of Emotional vulnerability).

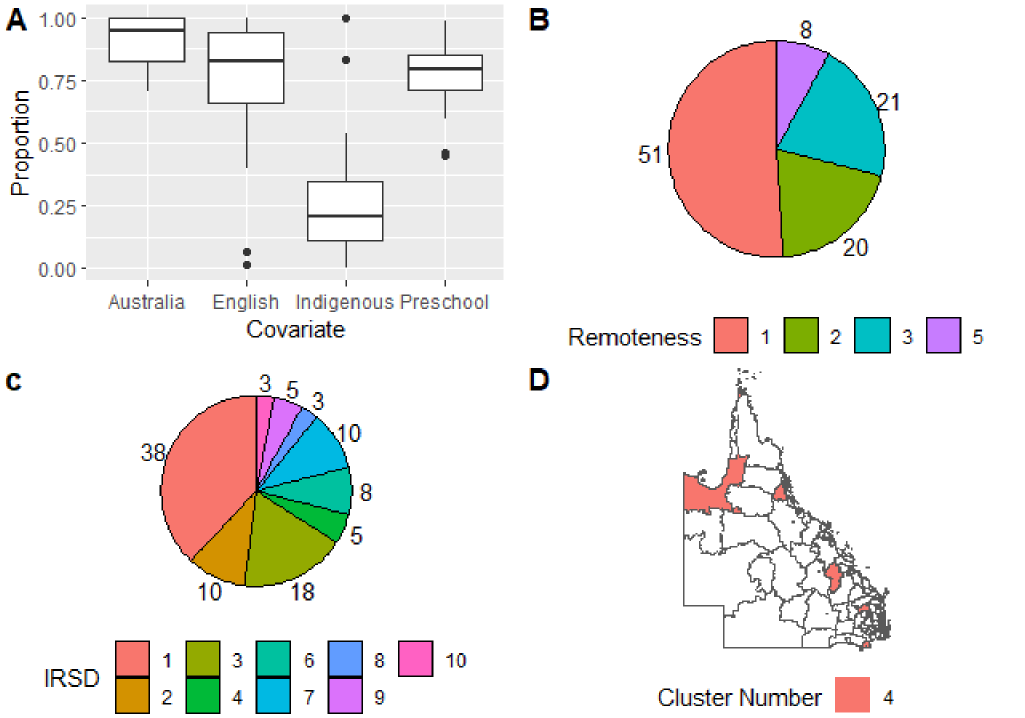

Figure 6: Fourth cluster for Emotional using K-means, SA2's size=39 (highest SA2 proportion of Emotional vulnerability).

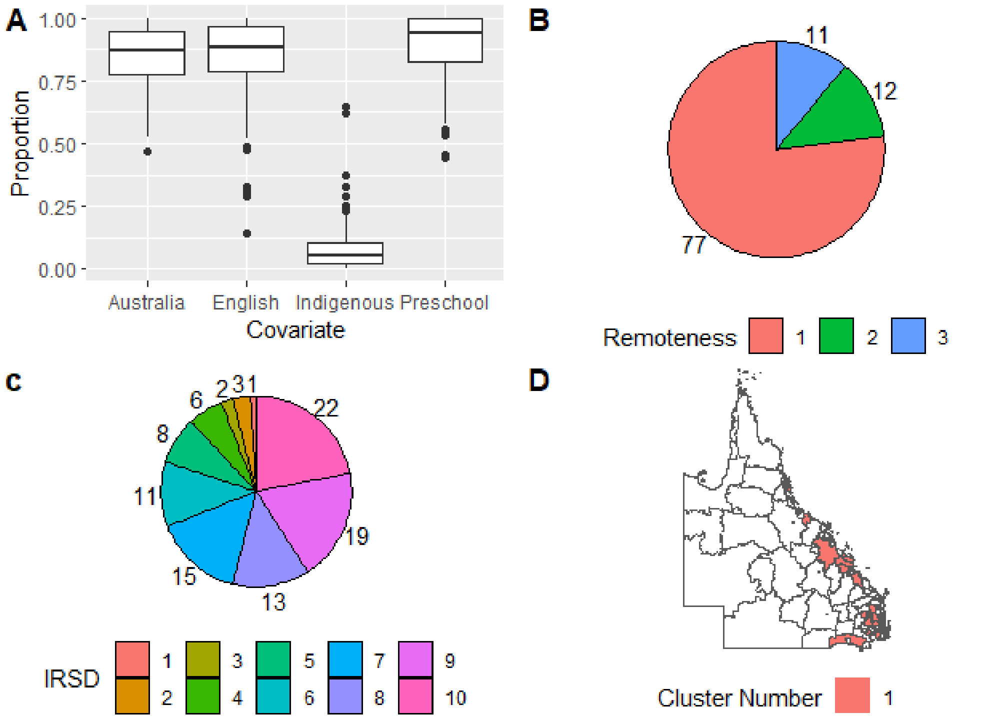

Figure 7: First cluster for Language using K-means, SA2's size=162 (lowest SA2 proportion of Language vulnerability).

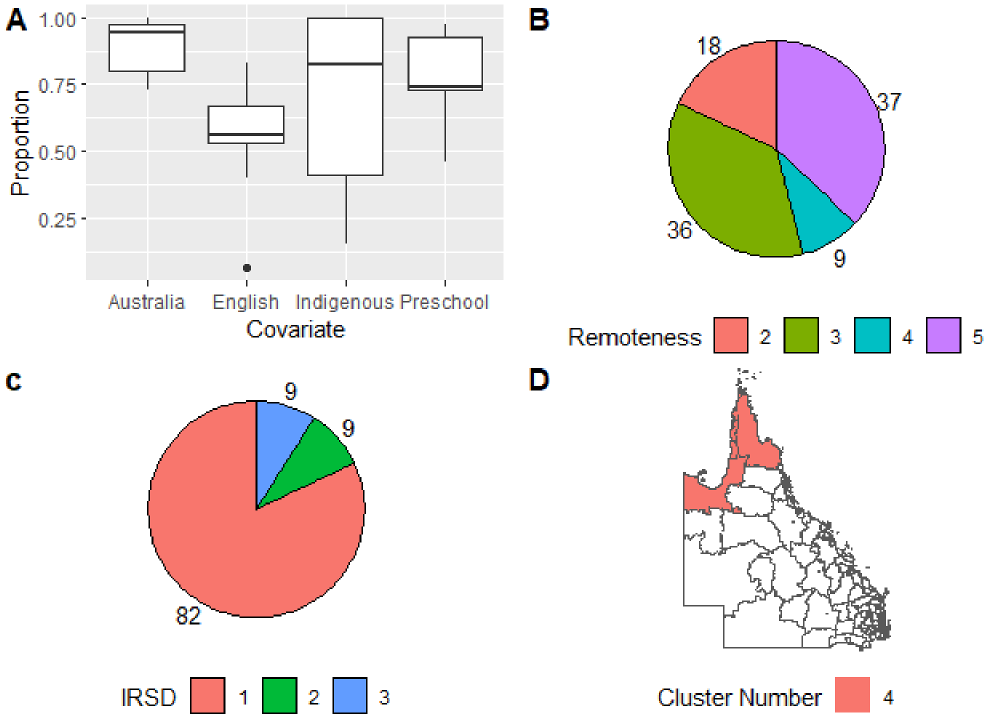

Figure 8: Fourth cluster for Language using K-means, SA2's size=37, (highest SA2 proportion of Language vulnerability).

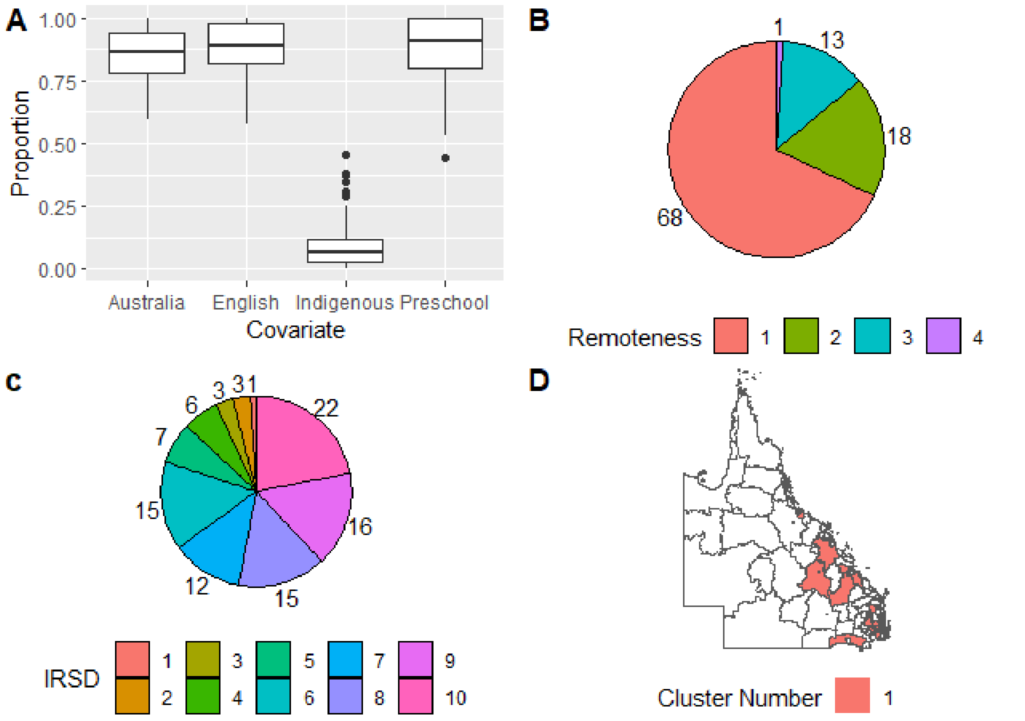

Figure 9: First cluster for Communication using K-means, SA2's size=152 (lowest SA2 proportion of Communication vulnerability).

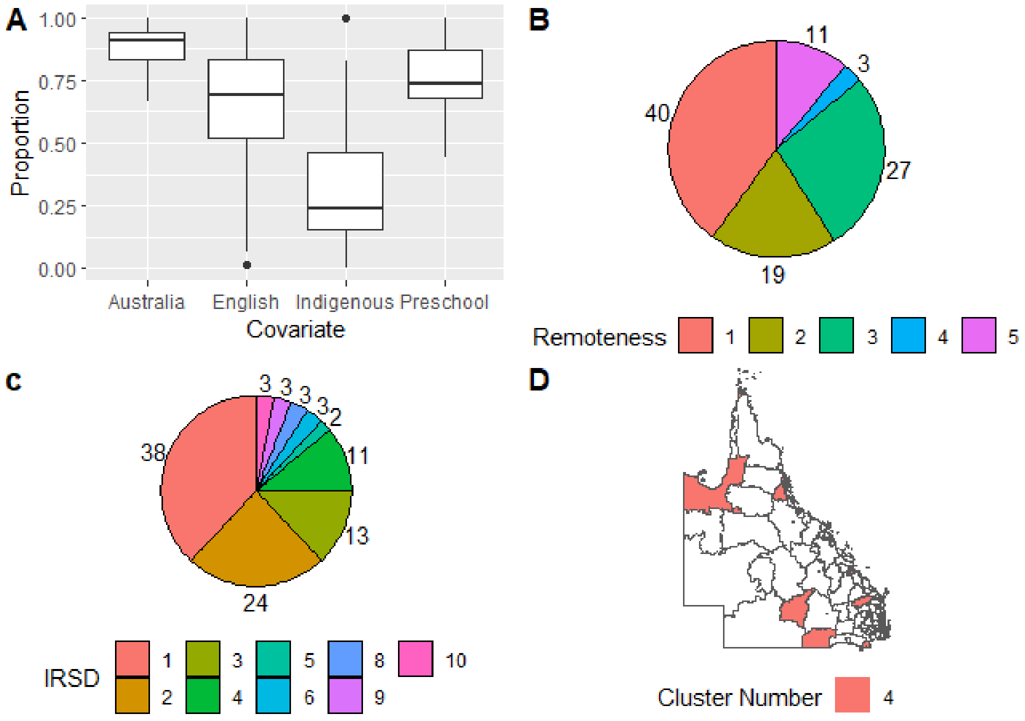

Figure 10: Fourth cluster for Communication using K-means, SA2's size=46 (highest SA2 proportion of Communication vulnerability).

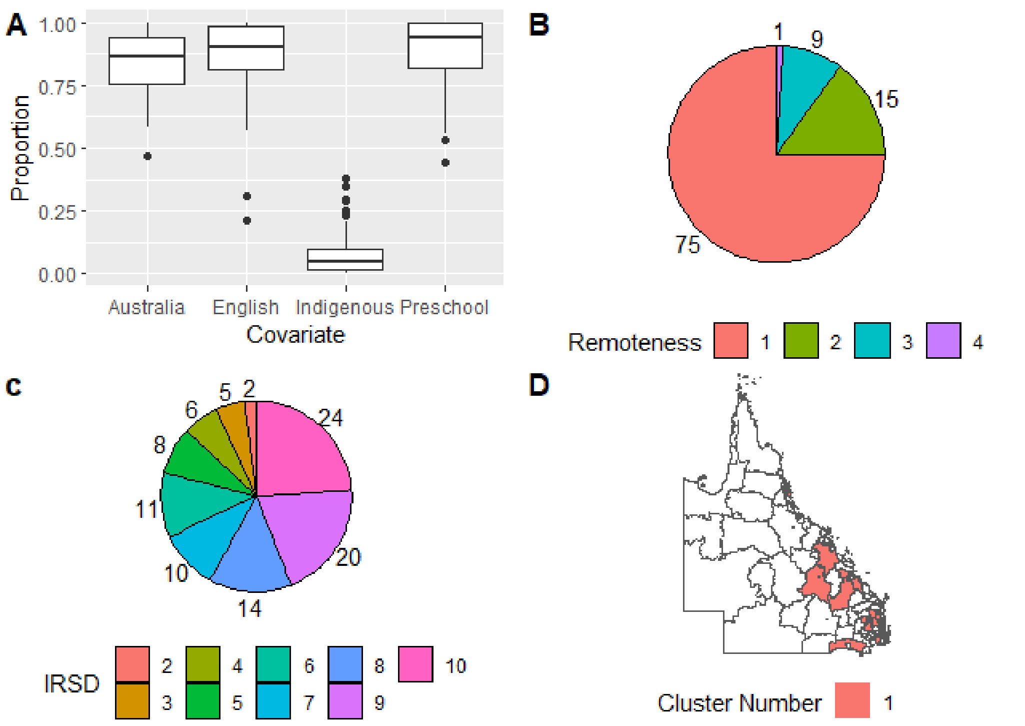

Figure 11: First cluster for Vuln 1 using K-means, SA2's size=101 (lowest SA2 proportion of Vulnerability on two or more domain/s).

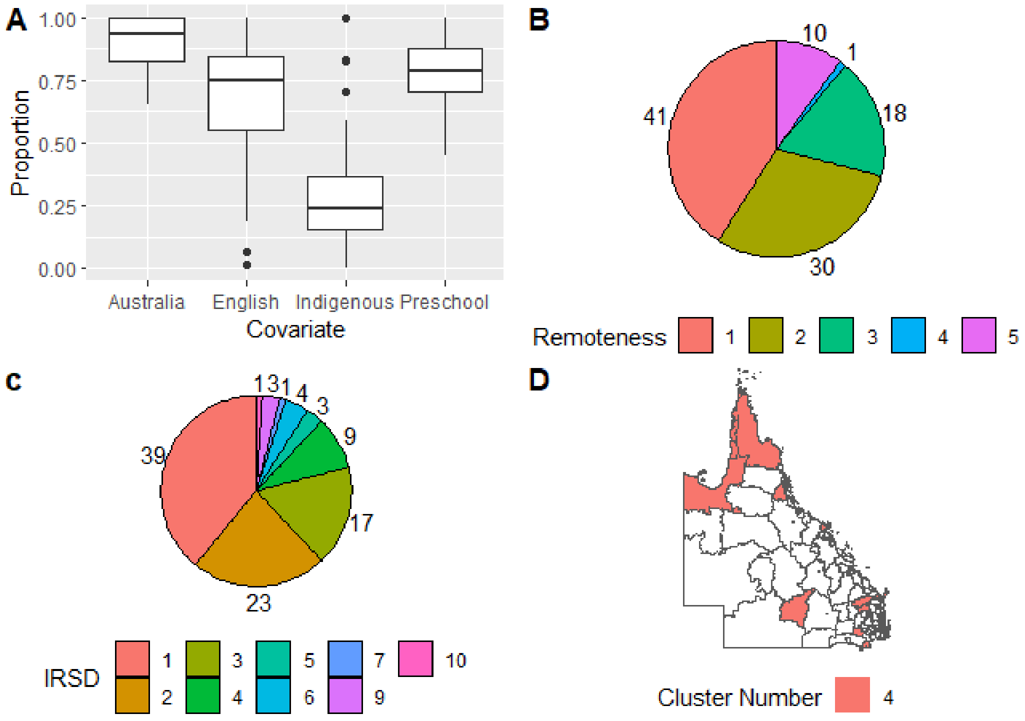

Figure 12: Fourth cluster for Vuln 1 using K-means, SA2's size=71 (highest SA2 proportion of Vulnerability on one or more domain/s).

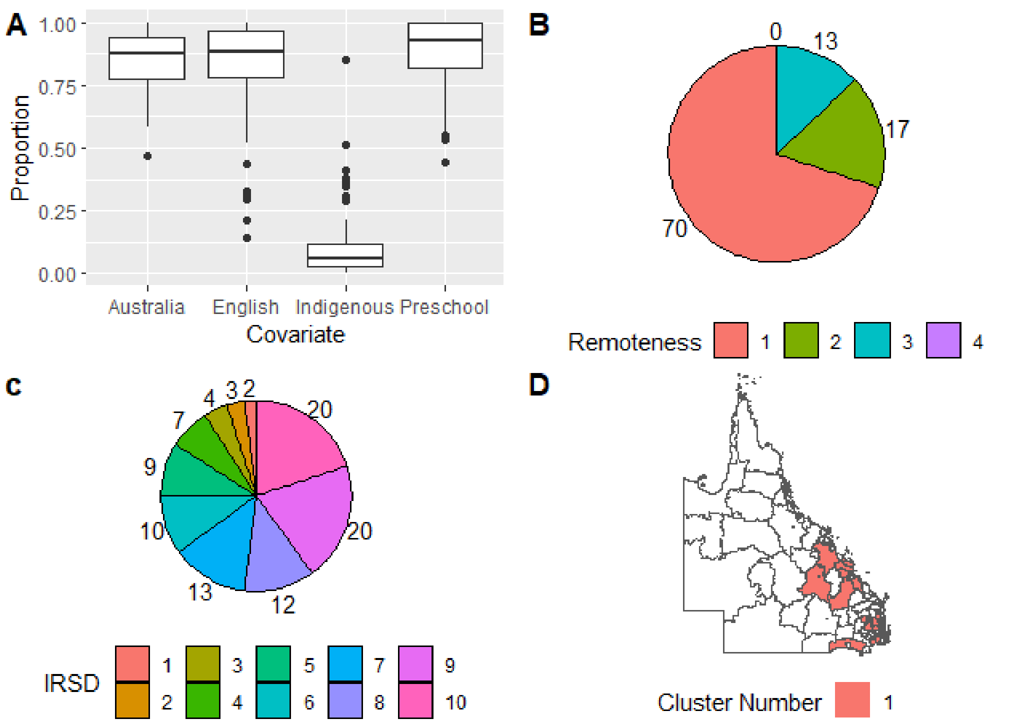

Figure 13: First cluster for Vuln 2 using K-means, SA2's size=162 (lowest SA2 proportion of Vulnerability on two or more domains).

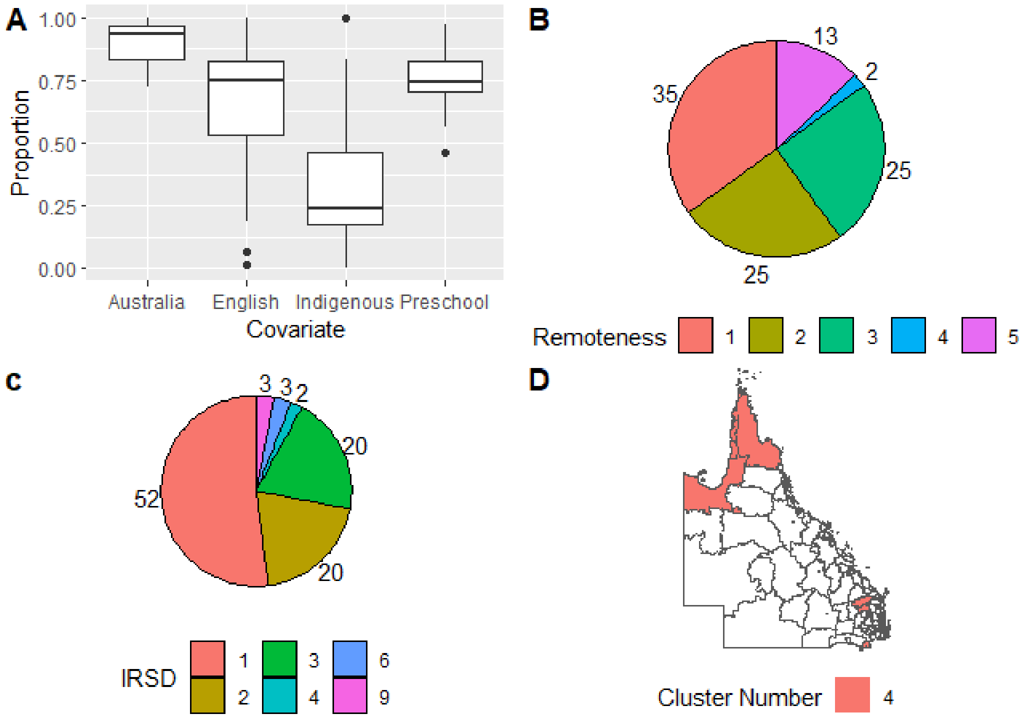

Figure 14: Fourth cluster for Vuln 2 using K-means, SA2's size=40 (highest SA2 proportion of Vulnerability on two or more domains).

References

[1] P. Moraga. Spatialepiapp: A shiny web application for the analysis of spatial and spatio-temporal disease data. *Spatial and Spatio-Temporal Epidemiology*, 23:47–57, 2017.

[2] P. Moraga. *Geospatial Health Data: Modeling and Visualization with R-INLA and Shiny*. CRC Press, 2019.
